# Young children's capacity to imagine and prepare for certain and uncertain future outcomes

**DOI:** 10.1371/journal.pone.0202606

**Published:** 2018-09-04

**Authors:** Jonathan Redshaw, Talia Leamy, Phoebe Pincus, Thomas Suddendorf

**Affiliations:** School of Psychology, University of Queensland, St Lucia, Queensland, Australia; University of Portsmouth, UNITED KINGDOM

## Abstract

The current study used a minimalist paradigm to examine young children’s capacity to imagine and prepare for certain and uncertain immediate future outcomes. In a counterbalanced order, 2.5-year-old children (*N* = 32) completed twelve trials each of two tasks: (1) the forked tube task, which assessed their ability to cover two possible tube exits to ensure they would catch a single target with an uncertain future trajectory, and (2) the double tube task, which assessed their ability to cover two separate tube exits to ensure they would catch two targets with certain future trajectories. Even though the optimal preparatory action was the same across both tasks, children were much more likely to spontaneously and consistently demonstrate this action in the double tube task than the forked tube task. Children’s responses were unaffected by the number of targets seen in the demonstration phase, and instead appeared to be based on the particular contingencies of each apparatus. These results are consistent with the possibility that young children specifically struggle to imagine and prepare for mutually exclusive versions of uncertain future events.

## Introduction

The capacity to imagine and prepare for future events is central to complex human behaviour [1]. Because many future events are uncertain, however, we often decide to prepare for possible outcomes that we know may not eventuate [2–3]. Such preparatory behaviour ostensibly serves to maximise the long-term benefits to an individual, even at the risk of sometimes over-preparing. A person going on a hike, for example, may consider packing sunscreen, sunglasses, and a picnic rug in preparation for sunny weather, but also an umbrella, raincoat, and plastic bags in preparation for rainy weather. If the weather happens to be either sunny or rainy the whole time, then packing two sets of preparatory items would turn out to have been unnecessary; but frequently it is wise to prepare for multiple possibilities even if it does mean carrying extra weight or incurring some other costs. This ability to consider alternative future possibilities under conditions of uncertainty–and to prepare accordingly–has recently received interest in many domains of the behavioural sciences (e.g., [4–8]).

In developmental psychology, a large body of research demonstrates that children begin to think about the future and prudently prepare for upcoming events during the preschool years (for reviews, see [9–13]). These studies, however, have almost exclusively employed tasks assessing children’s capacity to anticipate future events with only one plausible outcome. Typically, a task will require children to select a correct solution (from among several options) to a highlighted future problem, and the participants’ performance is then compared to that expected by chance (see, e.g., [14–20]). Therefore, although these paradigms provide valuable insight into the development of children’s basic capacity for foresight, they fail to capture the inherent uncertainty that is quintessential to adult conceptions of the future [3].

Until recently, only two studies had assessed children’s capacity to prepare for multiple, mutually exclusive outcomes of an uncertain future event. Beck, Robinson, Carroll, and Apperly [21] gave 3- to 5-year-old children a task where they had to place mats to protect a toy mouse falling down a series of slides. In one condition, the mouse could fall down one of two sides of a forked slide, such that children had to place mats at the bottom of both sides to guarantee the mouse’s safety. Less than half of the children, however, did place mats at the bottom of both sides. Robinson, Rowley, Beck, Carroll, and Apperly [22] found a similar pattern of results in a task where 4- to 6-year-old children had to strategically place two trays in order to catch a block that could fall from one of two chutes. Each of these studies, however, included a complicated set of verbal instructions describing the link between the preparatory behaviour and the future event, and they also included practice phases where the optimal response was demonstrated to the children. It therefore remained possible that these studies either underestimated or overestimated children’s capacity to spontaneously and insightfully prepare for multiple possible versions of the future.

**Fig 1:**
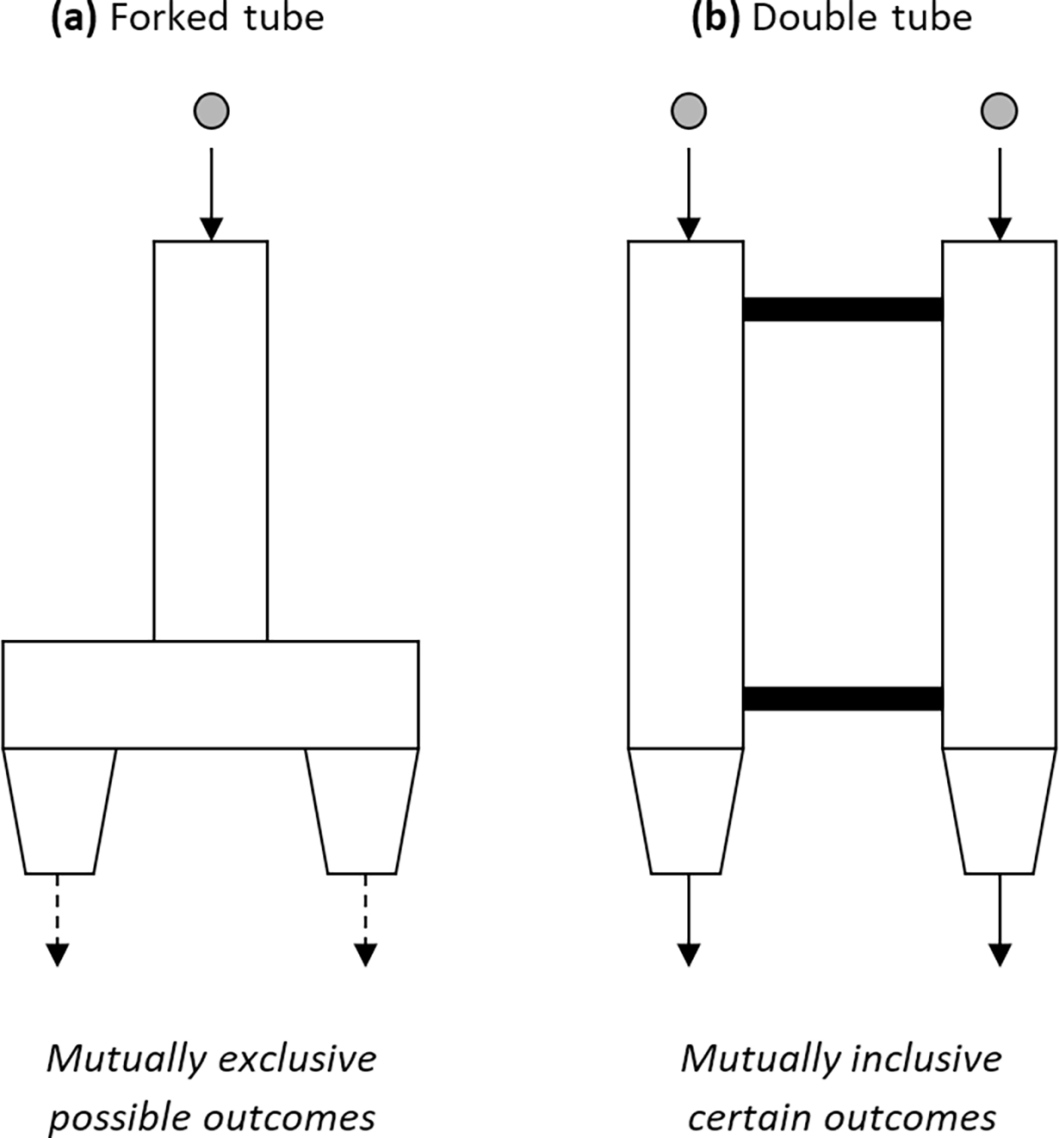
Schematic representation of **(a)** the forked tube task, where one ball is dropped into the top opening and can exit from either bottom opening, and **(b)** the double tube task, where two balls are simultaneously dropped into the top openings and must exit from both bottom openings. For both tasks the optimal behavioural response is to cover both bottom exits (with one hand each) when attempting to catch the ball/s. Only in the forked tube task, however, are children required to imagine and prepare for an uncertain future event with mutually exclusive possible outcomes.

Inspired by these earlier paradigms, Redshaw and Suddendorf [23] developed a minimalist task with essentially no linguistic demands and no demonstration of how to pass. Children aged between 2 and 4 years were introduced to an inverted Y-shaped ‘forked tube’ with one opening at the top and two exits at the bottom (see Fig 1 for a schematic representation). The experimenter could drop a ball into the top of the tube and surreptitiously control which exit it would fall from (in a pseudorandom order). After an initial observation phase–in which they could see the ball fall but not catch it–children were given the opportunity to catch the ball for 12 trials, and each time they failed to do so it fell on a ramp and rolled away. The results showed that most 4-year-olds spontaneously and consistently prepared for both mutually exclusive outcomes from the first trial onwards, by placing a hand under each exit when attempting to catch the ball. All 2-year-olds and many 3-year-olds, on the other hand, covered only one exit on the first trial, and many of those that did eventually cover two exits regressed to covering one exit on subsequent trials. One interpretation is that most of the older children, but few of the younger children, possessed an understanding that single future events can have multiple possible outcomes.

The forked tube paradigm has numerous advantages for examining the basic cognitive capacity to imagine and prepare for mutually exclusive future outcomes. Firstly, given the lack of reliance on verbal instructions, the task can easily be administered to children from diverse cultures [24] and even non-human primates [23]. Secondly, given that the preparatory behaviour occurs in the same context as the eventual future event, failure cannot be attributed to problems with the separate capacity to imagine non-present spatial settings [8,13]. Furthermore, given that the unpredictability of the event is high (50% chance of each outcome occurring) and the cost of preparing for two alternatives instead of just one is trivial, failure is unlikely to be due to children assuming that one alternative is rare and not worth preparing for (as in many real-world situations). Nevertheless, as is often the case in developmental psychology, failure at the forked tube task is still difficult to interpret. Indeed, young children may neglect to show the optimal response for reasons unrelated to the uncertainty of the future outcome.

One possible explanation for young children’s poor performance is that, rather than being specifically limited in the capacity to imagine and prepare for mutually exclusive outcomes, they are more generally limited in their capacity to imagine and simultaneously prepare for *multiple* future outcomes. Future possibilities are sometimes mutually exclusive (as in the forked tube task), but they can also be mutually *inclusive* (e.g. the weather on a hike may include both sunny and rainy periods). Conceivably, young children may simply lack the working memory resources required to mentally entertain two possible outcomes (i.e., ball falling from the left and ball falling from the right), independent of the fact that these two outcomes are mutually exclusive. Another possibility is that young children may simply lack the general physical capacity to coordinate two hands in the way that the forked tube task requires. If so, then the task may not be particularly suitable for examining young children’s capacity to imagine and prepare for multiple future outcomes–even if it is suitable for older children. Finally, young children may simply be primed to use the same number of hands as the number of balls used during the demonstration phase of the task (i.e., use one hand because there is only one ball), instead of responding based on the particular contingencies of the tube itself.

Children’s failures on cognitive tasks are always difficult to interpret on their own, yet by examining differential patterns of success and failure across various conditions we can increasingly narrow down the possibilities. The current study aimed to address the three aforementioned alternative explanations for young children’s poor performance on the forked tube task, and thus shed further light on their capacities and limitations with regards to thinking about and preparing for uncertain future events.

### The current study

The participants in this study were 2.5-year-old children, who were chosen because they typically perform poorly at the forked tube task [23]. These children completed not only the original forked tube task but also a novel ‘double tube’ task (in a counterbalanced order). The double tube task involved an apparatus with two separate tubes attached together and running top to bottom, such that there were two top openings and two bottom exits (see Fig 1 for a schematic representation and comparison to the forked tube task). This apparatus had been previously used in a separate task examining children’s and apes’ capacity to prepare for a single uncertain event controlled by a social agent [25]. In the current double tube task, however, the experimenter dropped *two* balls simultaneously–one down each of the connected tubes–such that children had to cover both bottom exits to catch both balls. Thus, although the optimal preparatory behaviour required to pass this task (covering two exits) is exactly same as in the forked tube task, the cognitive processes involved in passing may be fundamentally different. Specifically, whereas the forked tube task was designed to assess a capacity to imagine and prepare for two mutually exclusive possibilities of a single uncertain future event, the double tube task is designed to assess a capacity to imagine and prepare for two mutually inclusive and certain future outcomes.

During the initial task demonstration phase, when the children could observe but not attempt to catch the targets, the experimenter dropped either one or two balls into each apparatus (counterbalanced across participants). This was to examine whether children’s responses were simply primed by the number of balls used during this phase, rather than by the particular contingencies of the apparatuses themselves. In the two-ball demonstration phase of the forked tube task, the balls were made to exit the same side of the tube simultaneously (i.e., with one ball directly trailing the other), such that the only difference between conditions was in the number of balls falling–and not in the exits that the balls fell from *per se*.

Essentially, the current study pitted the prevailing explanation for young children’s difficulty with the forked tube task–which we refer to as the *immature prospection hypothesis*–against three alternative explanations. If young children struggle on the forked tube task but not the double tube task, then this would be consistent with the immature prospection hypothesis, that (i) young children are specifically limited in their capacity to imagine and prepare for multiple, mutually exclusive future outcomes [23]. If, on the other hand, they struggle on both tasks, then it could be possible that they are merely limited in (ii) their general capacity to imagine and prepare for two future outcomes, regardless of the mutual exclusivity/inclusivity of these outcomes, and/or (iii) their general capacity to physically coordinate two hands in the way required by the tasks. Alternatively, if young children tend to cover one exit when they see one ball dropped in the demonstration phase but two exits when they see two balls dropped in the demonstration phase (and their responses to do not vary as a function of the tasks themselves), then this would suggest that (iv) they are primed to respond in a particular way by the actions of the experimenter. Although it is impossible to provide direct evidence *for* the immature prospection hypothesis–as it is effectively about the absence of a cognitive capacity–we can be more confident in its veracity by ruling out some likely alternatives.

## Method

### Participants

A total of 40 children were recruited for the study, 8 of whom were excluded from the final sample because of failure to complete both tasks or because of prompting from their parents regarding how to respond. The final sample consisted of thirty-two 2.5 year-olds (*M* age = 29 months and 16 days, *SD* = 1 month and 5 days; 12 boys and 20 girls). Because our main statistical analysis involved computing generalised chi-square values for each effect, we conducted a chi-square power analysis with an estimated large effect size (*w*) of 0.5 (see [26]). This power analysis was based on our likelihood of detecting main effects only, as we made no *a priori* predictions about interactions. Results of the analysis suggested that we had an 80.7% chance of detecting the hypothesised large difference between tube tasks, which we predicted based on the assumption that the tasks were measuring fundamentally different cognitive capacities [23].

The study was approved by the university’s School of Psychology ethical review board (clearance 16-PSYCH-4-13-JS), and guardians provided informed verbal and written consent for their children to participate.

### Materials

The single tube (used in the practice phase only) and forked tube were the same as those used in Redshaw and Suddendorf’s study [23], and the double tube was the same the one used by Suddendorf et al. [25]. All of these tubes were principally constructed out of PVC pipe fittings.

#### Single tube

The single tube consisted of a single cylindrical pipe (approximately 9cm in diameter and 50cm long) connected to a tapering fitting at the bottom, such that a child could place their hand entirely over the exit and ensure that they would catch a ball dropped into it.

#### Forked tube

The forked tube consisted of a cylindrical pipe (approximately 9cm in diameter and 50cm long) connected to an inverted T-shaped fitting at the bottom. Each side of the T section was connected to a 90° L-shaped fitting, and each L-shaped fitting was in turn connected to a tapering fitting, again such at a child could entirely cover the exit if they wished. The two bottom exits were located approximately 25cm apart, such that a child could easily cover both exits simultaneously. A funnel was fasted to the inside of the top of the tube, such that a ball dropped inside would fall in approximately the same place every time. Approximately 3cm underneath the funnel was a wooden platform, which could be rotated by turning a wingnut screw located on the outside of the tube (this screw was visible to the experimenter but not the participants). Underneath the wooden platform was a piece of cardboard running the length of the main tube, which separated the tube into two parallel compartments. Effectively, turning the wingnut screw and rotating the wooden platform gave the experimenter surreptitious control over which bottom exit a ball would fall from after it was dropped into the tube.

#### Double tube

The double tube consisted of two cylindrical pipes (each approximately 9cm in diameter and 50cm long) connected by two metal holdings that ensured the pipes remained parallel to each other. The bottom of each pipe was connected to a narrowing fitting, again such that a child could entirely cover each exit with their hand. As for the forked tube, the two exits were spaced approximately 25cm apart. Each pipe was also attached to an L-shaped metal fitting, such that the entire apparatus could be fastened with clamps to a metal white stand approximately 60cm high. This allowed the experimenter to simultaneously drop two balls into the parallel pipes without having to hold onto the apparatus. The stand remained on top of the ramp for all tube tasks, to ensure that testing conditions remained comparable across tasks.

#### Other

The balls dropped into the tube were colourful polybutadiene ‘bouncy balls’ approximately 3cm in diameter. For each task, the balls were dropped such that they would fall onto a ramp and roll away if they were not caught. This was accomplished by leaning a plywood ramp against a small wooden chair approximately 40cm high. Next to the ramp was a small bucket, in which children could place any balls they caught.

### Procedure

#### Practice phase (single tube)

Children and parents were invited into the experimental room, and parents were encouraged to sit on a seat at the opposite side of the room from the testing area (some parents sat behind their children to calm them). The children were encouraged to place their hands behind their back, and the experimenter (TL) dropped three balls consecutively into the single tube, each of which landed on the ramp and rolled away. The children were then encouraged to catch the ball, with the experimenter showing them how to place their hand directly over the single tube exit. Children were told to place any caught balls into the bucket beside them, and the practice trials continued until they had caught three balls consecutively (usually on the first three attempts).

All children completed this practice phase first. Half of them then completed the forked tube task before the double tube task, whereas the other half completed these phases in the opposite order. Half of the children from each task-order group were in the *one-ball demonstration group*, and the other half were in the *two-ball demonstration group*.

#### Forked tube phase

The experimenter asked the children to place their hands behind their back once more, before commencing the six observation trials. For the *one-ball demonstration group*, the experimenter touched or turned the wingnut screw before each trial, forcing the balls to come out of the exits in the following pseudorandom order: *right*, *left*, *left*, *right*, *left*, *right* (from the experimenter’s perspective). For the *two-ball demonstration group*, the experimenter dropped two balls into the tube at the same time, using the wingnut screw to force both balls to exit the same side of the tube in the same pseudorandom order. The purpose of this phase was to enable children to observe the contingencies of the apparatus prior to having the opportunity to respond.

After the observation phase, the experimenter told the children that they could try to catch the balls again (without mentioning the opportunity to cover both exits), and that if they caught lots of balls they would be rewarded with stickers. For children in both demonstration groups, the experimenter touched or turned the wingnut screw before each of the twelve test trials, forcing a single ball to exit in the following pseudorandom order: *right*, *left*, *left*, *right*, *left*, *right*, *right*, *left*, *right*, *left*, *left*, *right* (from the experimenter’s perspective). Again, children were encouraged to place caught balls into the bucket.

#### Double tube phase

The experimenter again asked the children to put their hands behind their back, before commencing six observation trials. For the *two-ball demonstration group*, the experimenter dropped two balls simultaneously (one down each opening) on all observation trials, causing them to land on the ramp and roll away from the children. For the *one-ball demonstration group*, the experimenter dropped one ball down one of the adjacent tubes in the following pseudorandom order: *left*, *right*, *right*, *left*, *right*, *left* (from the experimenter’s perspective). Again, the purpose of this phase was to enable children to observe the contingencies of the apparatus before having the opportunity to respond.

The experimenter then told the children that they could try to catch the balls (again without mentioning the opportunity to cover both exits), and that if they caught lots of balls then they would be rewarded with stickers. Twelve test trials immediately followed, with the experimenter always dropping two balls simultaneously (one down each opening; for both demonstration groups) and asking the children to place any caught balls into the bucket. All children were rewarded with stickers once both of the tasks were completed.

### Coding

Across both the forked and double tube phases, children were considered to pass a trial if they were at least partially covering two exits (with one hand each) as the ball/s fell. Children who covered a single exit (or, very rarely, no exits) were considered to fail. Additionally, children were classified into one of four categories based on their response patterns across each tube task: (1) those who covered two exits on the first trial and all subsequent trials, (2) those who failed to cover two exits on the first trial, but did cover two exits at some stage and maintained that response across all subsequent trials, (3) those who covered two exits on at least one trial (first or otherwise) but regressed to covering only one exit on at least one subsequent trial, and (4) those who failed to cover two exits on any trial (see [23]). Data and statistical syntax files are in the Supporting Information (S1–S3 Files).

## Results

### Trial-by-trial analysis

Children’s performance across all trials was entered into a series of Generalised Estimating Equations (GEE) analyses containing effects nested within the following full factorial model: Tube (forked vs. double) x Trial (12 trials each tube) x Order Group (forked first vs. double first) x Demonstration Group (one ball vs. two balls). Models were compared and selected on the basis of lowest QIC value (see [27]). The best fitting model did not contain any effects involving the Demonstration Group variable, suggesting that children were not primed to respond based on the number of balls used in the demonstration phase of each task. Indeed, across both tasks and all trials, children in the one-ball demonstration group covered two exits on 285 out of 384 trials, and children in the two-ball demonstration group covered two exits on 261 out of 384 trials. We have therefore collapsed across the two levels of Demonstration Group for all of the following effects and figures.

**Fig 2:**
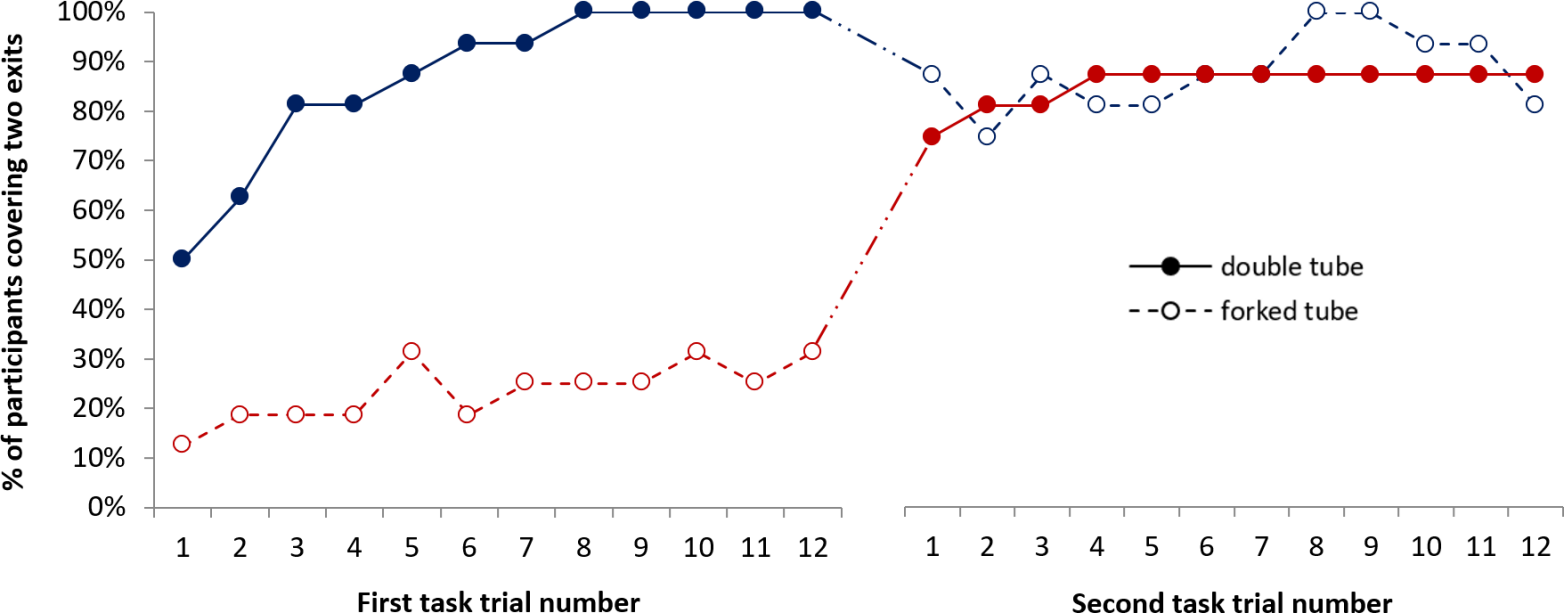
Percentages of 2.5-year-olds covering two exits across all 12 trials of the forked and double tube tasks. The red line represents the group who completed the forked tube task first, whereas the blue line represents the group of children who completed the double tube task first.

The best fitting model contained a significant main effect of Tube, Generalised Score χ^2^ (1) = 6.79, *p* = .009, which was qualified by a significant Tube x Order Group interaction, Generalised Score χ^2^ (1) = 10.07, *p* = .007. As seen in Fig 2, the two order groups showed differential patterns of responding across the tasks. The group that completed the forked tube task first (red line) performed significantly poorer on this task than on the double tube task, Wald χ^2^ (1) = 16.92, *p* < .001, whereas the group that completed the double tube task first (blue line) showed no difference in performance between tasks, Wald χ^2^ (1) = 0.01, *p* = .918.

Alternatively, when considering only the first task each group completed (i.e., trials 1–12), performance was significantly higher in the group that completed the double tube task than the group that completed the forked tube task, Wald χ^2^ (1) = 9.31, *p* = .002. When considering only the second task each group completed (i.e., trials 13–24), however, there was no significant difference in performance between groups, Wald χ^2^ (1) = 1.13, *p* = .287.

The best fitting model also contained a significant main effect of Trial, Generalised Score χ^2^ (1) = 14.70, *p* < .001, which was qualified by a significant Trial x Order Group interaction, Generalised Score χ^2^ (2) = 6.26, *p* = .012, suggesting that children’s average rate of change in performance across the trials of each tube task varied between the order groups.

### Overall performance pattern analysis

**Fig 3:**
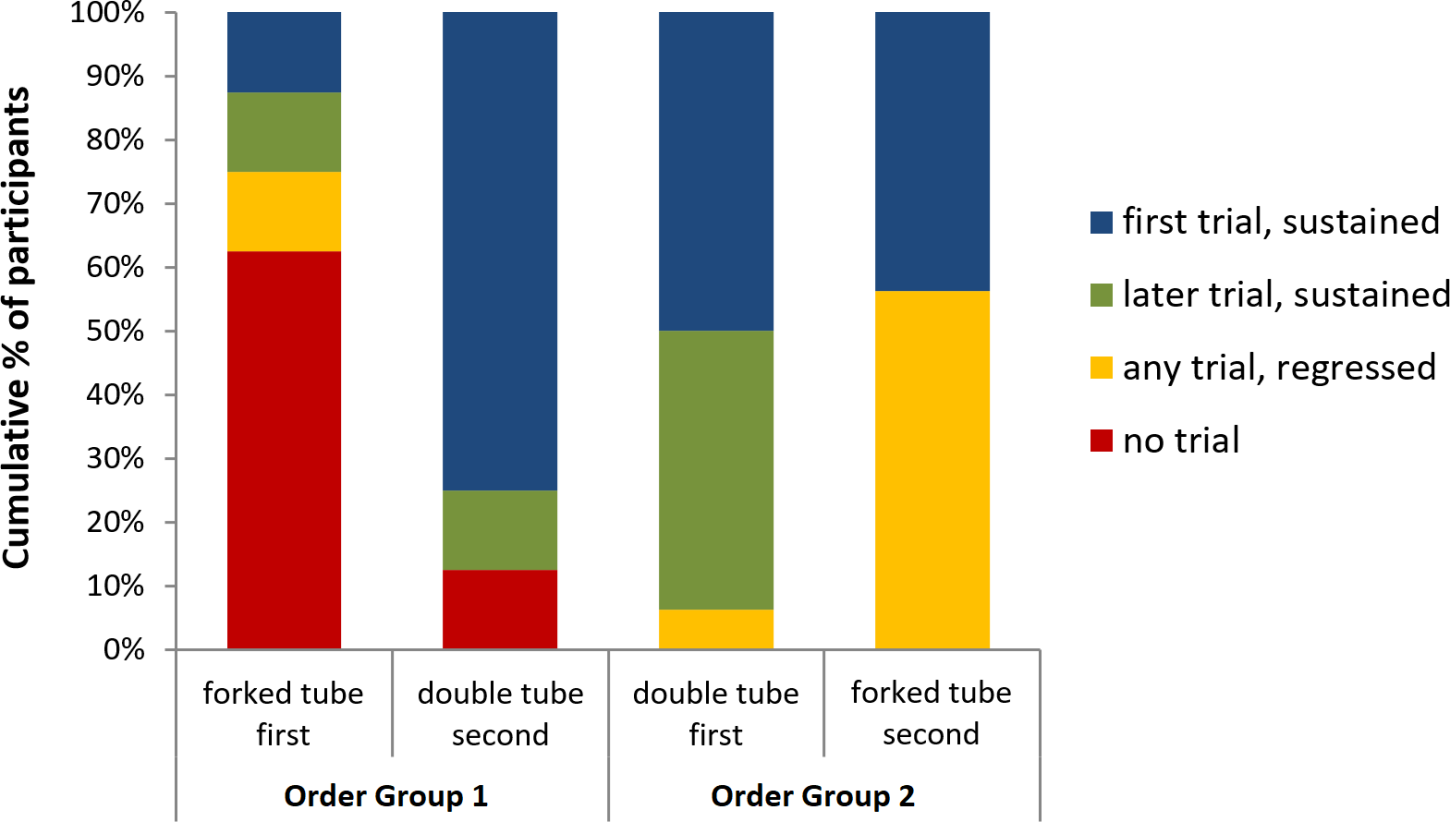
Cumulative percentages of 2.5-year-olds categorised according to when they covered two exits across all 12 trials of the forked and double tube tasks. The first two bars represent the group of children who completed the forked tube task first, whereas the second two bars represent the group of children who completed the double tube task first. See the coding section for detailed category definitions.

The performance patterns of the children in each Order Group across tasks are summarised in Fig 3.

### Group of children who completed the forked tube task first

In line with earlier findings [23], the 2.5-year-old children who completed the forked tube task first performed poorly, with 10 out of 16 failing to cover two exits on any trial, and only 2 out of 16 covering two exits on every trial. When these children subsequently completed the double tube task, however, the patterns were flipped, with most (12 out of 16) covering both exits on every trial and only 2 out of 16 failing to cover two exits at all. Among this group, there were significant changes between tasks in the proportions of children who never covered two exits, McNemar’s test, *p* = .008, and in the proportions of children who covered two exits on every trial, McNemar’s test, *p* = .002.

### Group of children who completed the double tube task first

The children who completed the double tube task first did very well, with half of them covering two exits on every trial and all 16 of them covering two exits on at least one trial. This group of children also performed relatively well on the subsequent forked tube task, with all of them again covering two exits on at least one trial. An important difference, however, lies in the proportions of children who, after covering two exits for the first time (on the first trial or otherwise), then regressed to covering only one exit on at least one subsequent trial (cf. yellow segments of the right two bars in Fig 3). Only 1 out of 16 children in this group showed such a response pattern during the double tube task, whereas 9 out of 16 showed it during the forked tube task, with the difference between these proportions significant, McNemar’s test, *p* = .008. Thus, although the overall trial-by-trial results suggested that children in this group performed similarly well on both tasks, the specific performance patterns show that they did indeed perform more poorly on the forked tube task.

## Discussion

The current study provides firm evidence against three alternative explanations for young children’s poor performance on the forked tube task, leaving the immature prospection hypothesis [23] as the most likely account of the data at present. The children who completed the forked tube task first performed poorly, and then sharply improved on the subsequent double tube task. This substantial increase in performance was likely due to the fact that these children no longer had to imagine and prepare for mutually exclusive future possibilities after switching tasks.

The children who completed the double tube task first, on the other hand, performed relatively well on both tasks. Still, they were significantly and substantially more likely to regress to covering only one exit on the forked tube task, even though they had extensive experience of catching balls by this stage. If children in this group were solving the forked tube task based on an insightful understanding that a single future event can have more than one possible outcome, then one would not expect the majority of them to fail trials after initially passing (cf. the 4-year-old children in [23]). Rather, it is more likely they were conditioned into covering two exits during the easier double tube task, with this response only weakly transferred across to the forked tube task–such that they still failed some of the trials. In sharp contrast, *none* of the children who completed the double tube task second regressed to covering a single exit after initially covering two (see Fig 3), suggesting there was no general propensity for children to regress simply as a function of performing so many consecutive trials.

In summary, children in both order conditions struggled on the forked tube task relative to the double tube task, which required the same physical response (i.e., cover two tube exits) but an alternative reasoning process (i.e., about two mutually inclusive and certain future outcomes, rather than two mutually exclusive and uncertain outcomes). Therefore, children’s trouble with the forked tube task could not be reduced to a general cognitive limitation with imagining and preparing for multiple outcomes. Given that children regularly covered both exits in the double tube version, there is also no sign that they have a general physical limitation with coordinating their hands. Finally, given that the number of balls used in the demonstration phase had no effect on children’s responses, the difference in performance across tasks could not be reduced to a simple response priming effect. One may contend that the lack of statistical difference between demonstration conditions was due to power limitations arising from the relatively small sample size. The descriptive data, however, showed no hint of this, with children in the one-ball demonstration condition actually covering two exits more (though not significantly so) than children in the two-ball demonstration condition. Critically, even given the small sample size, we were able to detect a large and highly significant difference between tube tasks (dependent on task order).

If young children are indeed specifically limited when it comes to imagining and preparing for mutually exclusive future possibilities, then one might ask which components of foresight they are lacking [8,13]. The participants in the current studies, after all, did not seem limited in their ability to predict an immediate future event *per se*, given that they covered at least one exit (and thus prepared for at least one future possibility) on nearly every trial across tasks. One possibility is that many young children lack the capacity to form *metarepresentations* [28–29], and therefore cannot reflect on the fact that their representations of future events can be incorrect [2,30]. A child could pass the forked tube task, for example, by first representing a single possible outcome (e.g., ball comes from the left exit), then recognising that this representation could be incorrect and preparing for the alternative possibility as well (i.e., ball comes from the right exit). In the double tube task, however, a child only has to entertain a simple representation of two balls exiting both sides simultaneously, without metarepresenting the relation between that representation and the actual future event. If this hypothesis is correct, then one might expect future studies to find children’s performance on the forked tube task (but not the double tube task) to be related to their performance on explicit false belief tasks (e.g., [31]), which are similarly thought to invoke a metarepresentational capacity [28].

Future research may also wish to consider comparing performance on the forked and double tube tasks in non-human primates. Initial results suggest that great apes may struggle to consistently pass the forked tube task and related tasks [23,25], but as for young children there are several possible explanations for these failures. An important consideration for future studies, however, is the length of the inter-trial interval. The previous study with the forked tube included only a very short interval [23], although the apes continued to perform poorly even when the experimenter forced the food reward to fall from whatever exit was uncovered (such that they needed to cover two exits to receive any food at all). If a very short interval was also used in the *double* tube task, however, then apes may not be motivated to cover both exits simply because they would be guaranteed to continually receive food rewards by covering only one exit on all trials. Thus, we recommend that such studies increase the inter-trial interval–in both the forked and double tube tasks–so that subjects would be highly motivated to catch all available rewards. Similarly, we suggest that studies maximise the subjective value of the food reward and only test apes when they are hungry.

In conclusion, our results are consistent with the hypothesis that young children are limited in the specific cognitive capacity to imagine and prepare for mutually exclusive possible future outcomes, rather than being limited in the general cognitive capacity to imagine and prepare for multiple outcomes or the general physical capacity to coordinate two hands. Research in this area is still in its infancy, and our minimalist paradigm lends itself to many opportunities for further studies in developmental psychology and beyond.

## Supporting information

S1 File SPSS raw data. (SAV)

S2 File SAS data file for analysis. (SAS7BDAT)

S3 File SAS syntax file for analysis. (TXT)
